# Interplay between integrins and cadherins to control bone differentiation upon BMP-2 stimulation

**DOI:** 10.3389/fcell.2022.1027334

**Published:** 2023-01-04

**Authors:** Anne Valat, Laure Fourel, Adria Sales, Paul Machillot, Anne-Pascale Bouin, Carole Fournier, Lauriane Bosc, Mélanie Arboléas, Ingrid Bourrin-Reynard, Amy J. Wagoner Johnson, Franz Bruckert, Corinne Albigès-Rizo, Catherine Picart

**Affiliations:** ^1^ Grenoble Institute of Engineering, CNRS UMR 5628, LMGP, Grenoble, France; ^2^ U1292 Biosanté, INSERM, CEA, CNRS EMR 5000 Biomimetism and Regenerative Medicine, University Grenoble Alpes, Grenoble, France; ^3^ U1209 Institut for Advanced Biosciences, CNRS 5309, University Grenoble Alpes, La Tronche, France; ^4^ Department of Mechanical Science and Engineering, University of Illinois at Urbana-Champaign, Urbana, IL, United States; ^5^ Carle Illinois College of Medicine, Urbana, IL, United States; ^6^ Carl R. Woese Institute for Genomic Biology, Urbana, IL, United States; ^7^ Institut Universitaire de France (IUF), Paris, France

**Keywords:** osteoblast differentation, BMP-2 (bone morphogenetic protein-2), adhesion receptors, integrins, cadherins, extracellular matrix (ECM)

## Abstract

**Introduction:** Upon BMP-2 stimulation, the osteoblastic lineage commitment in C2C12 myoblasts is associated with a microenvironmental change that occurs over several days. How does BMP-2 operate a switch in adhesive machinery to adapt to the new microenvironment and to drive bone cell fate is not well understood. Here, we addressed this question for BMP-2 delivered either in solution or physically bound of a biomimetic film, to mimic its presentation to cells *via* the extracellular matrix (ECM).

**Methods:** Biommetics films were prepared using a recently developed automated method that enable high content studies of cellular processes. Comparative gene expressions were done using RNA sequencing from the encyclopedia of the regulatory elements (ENCODE). Gene expressions of transcription factors, beta chain (1, 3, 5) integrins and cadherins (M, N, and Cad11) were studied using quantitative PCR. ECM proteins and adhesion receptor expressions were also quantified by Western blots and dot blots. Their spatial organization in and around cells was studied using immuno-stainings. The individual effect of each receptor on osteogenic transcription factors and alkaline phosphatase expression were studied using silencing RNA of each integrin and cadherin receptor. The organization of fibronectin was studied using immuno-staining and quantitative microscopic analysis.

**Results:** Our findings highlight a switch of integrin and cadherin expression during muscle to bone transdifferentiation upon BMP-2 stimulation. This switch occurs no matter the presentation mode, for BMP-2 presented in solution or *via* the biomimetic film. While C2C12 muscle cells express M-cadherin and Laminin-specific integrins, the BMP-2-induced transdifferentiation into bone cells is associated with an increase in the expression of cadherin-11 and collagen-specific integrins. Biomimetic films presenting matrix-bound BMP-2 enable the revelation of specific roles of the adhesive receptors depending on the transcription factor.

**Discussion:** While β3 integrin and cadherin-11 work in concert to control early pSMAD1,5,9 signaling, β1 integrin and Cadherin-11 control RunX2, ALP activity and fibronectin organization around the cells. In contrast, while β1 integrin is also important for osterix transcriptional activity, Cadherin-11 and β5 integrin act as negative osterix regulators. In addition, β5 integrin negatively regulates RunX2. Our results show that biomimetic films can be used to delinate the specific events associated with BMP-2-mediated muscle to bone transdifferentiation. Our study reveals how integrins and cadherins work together, while exerting distinct functions to drive osteogenic programming. Different sets of integrins and cadherins have complementary mechanical roles during the time window of this transdifferentiation.

## Introduction

The functional interactions between muscle and bone occur through growth factors and cytokines. This inter-organ communication is required both for maintenance of tissue homeostasis and for regeneration of bone tissue (Tsuji et al., 2006; Yu et al., 2010a; Glass et al., 2011; Rosen, 2011). Following bone injury, muscle-derived stem cells are activated during the inflammatory phase of repair (Colnot et al., 2003; Glass et al., 2011; Wang et al., 2013; Abou-Khalil et al., 2014). Satellite muscle cells can differentiate into osteoblasts and chondrocytes *in vitro* and *in vivo* (Asakura et al., 2001; Morrison et al., 2006; Liu et al., 2011; Cairns et al., 2012; Abou-Khalil et al., 2015). Skeletal muscle produces osteogenic-related factors such as insulin-like growth factor 1 (IGF1) and fibroblast growth factor 2 (FGF2) (Hamrick et al., 2010). In particular, skeletal stem cells are recruited by growth factors, including bone morphogenetic proteins (BMPs) at the fracture site, as well as by inflammatory and bone cells (Tsuji et al., 2006; Yu et al., 2010a; Rosen, 2011), in order to contribute to bone regeneration (Bosch et al., 2000; Wright et al., 2002; Liu et al., 2011). However, the involvement of BMPs in the signaling pathways responsible for the muscle-bone transdifferentiation process is still unclear.

BMPs are members of the transforming growth factor β (TGF-β) superfamily that control osteoblast proliferation and differentiation and induce ectopic bone formation *in vivo* when implanted into muscle tissue (Heldin et al., 1997; ten Dijke et al., 2000; Bouyer et al., 2016). BMP-2, BMP-4 and BMP-7 are key growth factors for normal bone development in vertebrates and are able to alter the C2C12 mesenchymal pluripotent cell lineage from the myogenic to the osteogenic phenotype (Akiyama et al., 1997; Yamamoto et al., 1997; Crouzier et al., 2011a). BMPs are recognized through heterodimeric complexes of transmembrane type I and type II Ser/Thr kinase receptors that then propagate signals through the SMAD pathway. SMAD proteins play a critical role in mediating BMP-induced signals from the cell surface to the nucleus; heterodimeric SMAD complexes function as effectors of BMP signaling by regulating transcription of specific genes (Massague and Wotton, 2000). Besides its role in osteoblastic differentiation, BMP-2 appears to control cytoskeletal rearrangement and cell migration, suggesting a role in mechanotransduction (Gamell et al., 2008; Kopf et al., 2014). We have shown that BMP-2 presented in a matrix-bound manner controls cell fate by inducing bone differentiation *in vitro* and *in vivo* (Crouzier et al., 2009; Crouzier et al., 2011b; Sales et al., 2022).

To achieve the correct tissue architecture during morphogenesis, cells must interact with each other and with the extracellular matrix (ECM). These interactions are mediated by two classes of adhesion receptors, namely the integrins for cell/matrix interactions (Berrier and Yamada, 2007) and the cadherins for cell/cell interactions (Weber et al., 2011), which are mechanically interconnected to drive tissue morphogenesis and to maintain tissue integrity (Chen and Gumbiner, 2006; Berrier and Yamada, 2007; Weber et al., 2011; Mui et al., 2016).

Our team has developed a biomaterial that enables the presentation of growth factors in a so-called “matrix-bound” manner, i.e., presented by the matrix (Crouzier et al., 2009). This presentation mode favors the interactions between the growth factors and their receptors by locally concentrating the growth factors at the basal side of the cells at the cell-matrix interface (Crouzier et al., 2011a), where adhesion receptors can also come into play. We have previously shown that BMP receptors and β3 integrins cooperate and converge to couple cell adhesion and migration to cell differentiation by controlling the early steps of cell spreading and SMAD signaling (Fourel et al., 2016).

Given the complex interplay between changes in the biochemical properties of the extracellular matrix during the time frame of muscle-osteogenic transdifferentiation, and different expression in the integrin and cadherin receptors set involved in muscle and bone differentiation (Mayer, 2003; Marie et al., 2014), we addressed the question as to whether BMP-2 may induce a switch in the cell adhesion machinery in concert with osteoblast tissue formation. Here, we focused more specifically on the kinetic response of BMP-responsive skeletal progenitors, namely C2C12 myoblasts, to the microenvironment during their transformation to muscle or osteoblast cells depending on the presentation of BMP-2. We provide evidence that BMP-2 is sufficient to change the cell adhesion repertoire, i.e., integrins and cadherins, and extracellular matrix composition in a osteoblast tissue-specific manner. Our results show that different sets of integrins and cadherins may have complementary functions during the time frame of muscle to osteoblast transdifferentiation. During the differentiation into osteoblasts, while integrins are working in concert with cadherins to control the early transcriptional activities, β1 integrin and cadherin 11 are more specifically dedicated to cell mechanics by both shaping octagonal cells and organizing their extracellular matrix.

## Material and methods

### Buildup of polyelectrolyte multilayer films, cross-linking and loading of BMP-2

Sodium hyaluronate (HA) (200,000 g/mol, Lifecore Biomedical, United States) and poly (l-lysine) (PLL) (20,000 g/mol, Sigma-Alrich, France) were dissolved in Hepes-NaCl buffer [20 mM Hepes pH 7.4, 0.15 M NaCl] at 0.5 mg/ml and 1 mg/ml respectively. For all experiments, polyelectrolyte multilayer films made of 12 layer pairs of (PLL/HA), were built on glass slides (VWR Scientific, France) or directly in 96-well plates (*Nunc*, Denmark) using a recently established automated process, as previously described (Machillot et al., 2018). The first deposited layer, prior to (HA/PLL), was always a layer of poly (ethyleneimine) (70,000 g/mol, Sigma, France) at 3 mg/ml. After buildup, films were crosslinked following a previously-published protocol using 1-Ethyl-3-(3-Dimethylamino- propyl) carbodiimide (EDC, Sigma-Alrich*,* France) at 70 mg/ml and N-hydrosulfosuccinimide (sulfo-NHS, Sigma-Aldrich*,* France) at 11 mg/ml in a solution of 0.15 M NaCl at pH 5.5 (Richert et al., 2004). The stiffness of the polyelectrolyte films is about 400 kPa as measured by nanoindentation using an atomic force microscope (Schneider et al., 2006). For BMP-2 loading in the films, the films were first pre-equilibrated in 1 mM HCl and then loaded with human recombinant BMP-2 (Medtronic, France) in 1 mM HCl at 37°C for 1 h30. The loaded films were thoroughly washed at least five times in Hepes-NaCl buffer at pH 6.5 in order to keep only the physically-bound BMP-2, named hereafter bBMP-2 (Crouzier et al., 2009). Finally, they were sterilized for 20 min under UV light.

### Cell culture

**FIGURE 1:**
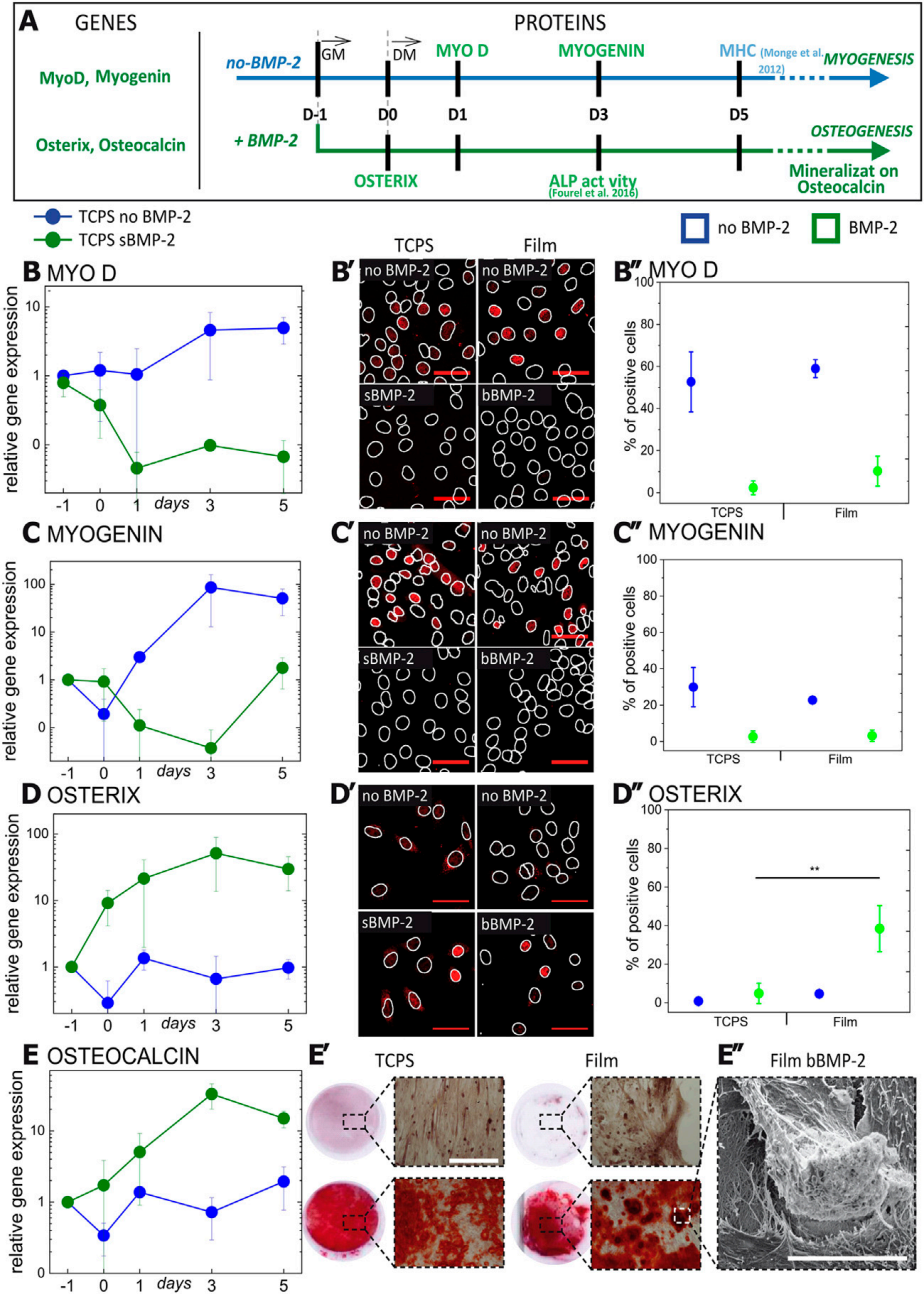
sBMP-2 and bBMP-2 drive the muscle to osteoblast transdifferentiation of C2C12 cells. Myogenic (blue) and osteogenic (green) differentiation of C2C12 cells assessed in the presence or absence of BMP-2. Cells were cultured on TCPS in the presence of soluble BMP-2 (sBMP-2). **(A)** Schematics indicating the different time points of the kinetic analysis and biological assays performed. C2C12 cells were seeded on TCPS as control with or without sBMP-2. 1 day after seeding in GM (D0), when cells were confluent, the medium was changed to DM. The medium was renewed at D3 and D5. **(B–E)** The kinetics of cell differentiation was assessed by measuring the gene expression of **(B)** MyoD and **(C)** myogenin for myogenic differentiation, and **(D)** Osterix and **(E)** Osteocalcin for osteogenic differentiation. The transcription factors **(B′)** Myo D, **(C′)** Myogenin, and **(D′)** Osterix were analyzed by immunofluorescence. Images correspond to D0. Nuclei were highlighted with white circle **(B′**,**C′,D′)**. Corresponding quantifications of the percentage of positive cells i.e., with a nuclear intensity of protein inside the nucleus above the threshold. Data are represented as box plots of three independent experiments **(B'',C'',D'')**. **(E′)** Alizarin red staining of calcium aggregates in the case of sBMP-2 for cells on TCPS and bBMP-2 for cells on films. **(E'')** SEM observation of the typical shape of calcium aggregates on bBMP-2-loaded films. Scale bar fluorescent images is 50 µm. Scale bar of alizarin red-stained samples is 400 µm. Scale bar of SEM image is 50 µm. Data are mean ± SD of three independent experiments. Statistical tests were done using non-parametric Kruskal–Wallis ANOVA (*p < 0.05; **p < 0.01).

C2C12 cells (ATCC^®^ CRL-1772™, <20 passages) were maintained in polystyrene flasks in a 37°C, 5% CO_2_ incubator and cultured in growth medium (GM [1:1 Dulbecco’s Modified Eagle Medium (DMEM):F12 medium (11320-074, Invitrogen), 10% fetal bovine serum (FBS, 10270-098, Invitrogen), 10 U/mL penicillin G and 10 μg/ml streptomycin (15140-122, Invitrogen). Cells were subcultured prior to reaching 60%–70% confluence. For all experiments, C2C12 cells were seeded at 30,000 cells/cm^2^ in GM until confluency (D0), when the medium was switched in differentiation medium (DM) (1:1 DMEM:F12 with 2% horse serum (HS from PAA Laboratories), 10 U/mL penicillin G and 10 μg/ml streptomycin as previously described (Ren et al., 2008). For the kinetics analysis of gene expression followed by qPCR, DM was refreshed at day 3 (D3) and day 5 (D5). Soluble BMP-2 (sBMP-2) was added in the GM at 600 ng/ml and refreshed at each change of medium until D5 (Figure 1A). Their translocations to the nucleus were observed by immunofluorescence at D0 for osterix, and at D1 and D3 for myogenin and MyoD, respectively. In addition, the ability of bBMP-2 films to induce later osteogenesis was confirmed by following the gene expression of Osteocalcin and by verifying cell mineralization.

### Comparative analyses of gene expression using RNA sequencing from UCSC genome browser

RNA sequencing (RNAseq) data were exported from the Encyclopedia of the regulatory elements (ENCODE). RNAseq analysis were performed for muscle cells (7 days-differentiated C2C12 - ENCSR000AHY and primary skeletal muscle myoblast (*Homo sapiens*) - ENCSR000CWN) and osteoblast cells (mean of responses of primary osteoblasts (*Homo sapiens*, adult 56-year female and adult 62-year male - ENCSR000CUF). The relative percentage of each of the adhesion receptors were calculated and compared for muscle and for bone tissues. The adhesion receptors with a relative expression <1% were excluded and not represented. Ratios comparing the relative expression in muscle and bone tissue were calculated by dividing the relative percentage of expression in each tissue.

### RNA isolation and reverse transcriptase-polymerase chain reaction (RT-qPCR)

The gene expression of the master transcription factors for myogenesis and osteogenesis and for the adhesion receptors integrins and cadherins were followed over time using RT-qPCR at D-1, D0, D1, D3, and D5. Total RNA was extracted from C2C12 myoblasts using a kit (Zymo research, Proteigene, France). Reverse transcription was done from 1 µg RNA using 5x iScript Reverse Transcription Supermix for RT-qPCR (170-8840, Invitrogen)*.* Real time-quantitative PCR was performed with a thermocycler MX4800P (Stratagène)*.* The reaction mix was composed of Master SYBR Green I mix (universal SYBR Green Supermix, 172-7272, Biorad) -containing dNTPs, Sso7d fusion polymerase, MgCl_2_, SYBR^®^ Green I, ROX normalization dyes-, 0.5 μM of each primer whose sequences are detailed in supplementary data (Supplementary Table S1) and 20-fold diluted cDNA. Primer efficiency was established by a standard curve using sequential dilutions of gene specific PCR fragments. According to MIQE guidelines (Bustin et al., 2009), results were normalized to the mean of the expression levels of the three more stable housekeeping genes, determined with GeNorm software, and expressed as a percentage of the control condition (the trio of housekeeping genes for each experiment are indicated in each legend).

### Immunofluorescence

Cells were fixed over night at 4°C in 3.7% formaldehyde (FA, F1635, Sigma-Aldrich) in PBS. Except for the imaging of the extracellular matrix proteins FN and COLL1, cells were permeabilized for 4 min in Tris Buffer Saline (TBS) (50 mM Tris, pH 7.4, 0.15 M NaCl) containing 0.2% Triton X-100. The samples were blocked in TBS containing 0.1% BSA for 1 h, and were incubated over night at 4°C in a primary antibody TBS solution containing 0.2% gelatin. The references of the antibodies are provided in the (Supplementary Table S2). The samples were then incubated in the solution of secondary antibodies diluted TBS containing 0.2% gelatin. F-actin was stained with rhodamine-phalloidin and nuclei were stained using Dapi. Samples were imaged either using an Axiovert 200 M inverted microscope (Zeiss, Germany) equipped with a CoolSNAP EZ CCD camera (Ropper Scientific, Evry, France) or with an In Cell Analyzer 2,500 imaging system (Molecular Devices, United States) (Sales et al., 2022).

### Quantitative image analysis

For image analysis, ImageJ and InCarta software (Molecular Devices, United States) was used to automatically segment nuclei, and then to quantify the intensity of the flurescence signal of the transcription factors (pSMAD1/5/9, RunX2 and Osterix) inside the nuclei and in the cytoplasm. The mean nuclear intensity was calculated and compared across groups. Cells with a nuclear intensity greater than or equal to 1.5 the mean nuclear intensity of negative control cells (i.e., in the absence of BMP-2) were considered positive. The percentage of positive cells was calculated for a given transcription factor.

For the quantification of FN organization, segmentation was performed with the trainable weka segmentation plugin (ImageJ) (Witten et al., 2011). Fast random forest was used as classifier with the following set of filters: Gaussian blur, hessian, membrane projection, mean, laplacian, sobel filter, difference of Gaussian, Variance, kuwahara. Three classes (“diffuse FN”, “fibrous FN” and “background”) were defined and the classifier was trained on labeled images. Following segmentation and classification, the area of each class of objects (fibrous FN, diffuse FN and background) was measured using the Particle Analyzer tool (ImageJ). The results are presented as the percentage of each of the two classes of fibronectin types (fibrous and diffuse) among the total amount of detected fibronectin.

### Mineralization: Alizarin red staining and imaging by scanning electron microscopy (SEM)

C2C12 were plated at 30,000 cells/cm^2^ in GM in a 24-well plate for 2 days after which the medium was changed to a mineralization medium (MM), made of αMEM (A10490-01, Invitrogen), 10% FBS (10270-098, Invitrogen), 10 U/mL penicillin G and 10 μg/ml streptomycin (15140-122, Invitrogen, France), 50 μg/ml ascorbic acid (A0278, Sigma-Aldrich, France) and 10 mM β-glycerophosphate (50,020*,* Sigma-Aldrich, France). For sBMP-2, BMP-2 was added to GM at 600 ng/ml and refreshed at each medium change. Cells were maintained in culture for 3 weeks, and the MM was changed every 2–3 days. For observations of calcium deposits, cells were fixed in 3.7% formaldehyde (FA, F1635, Sigma-Aldrich, France) in PBS for 20 min at room temperature. Calcium was stained by Alizarin Red S staining solution (40 mM Alizarin Red S, A5533*,* Sigma-Aldrich, pH 4.2) for 20 min at room temperature, followed by several rinsing steps with deionized water. Images were acquired using an Olympus BX41 microscope. For scanning electron microscopy (SEM) observations, cells were fixed with 2.5% glutaraldehyde in 0.1 M cacodylate buffer at pH 7.2 (C0250, Sigma-Aldrich, France) and dehydrated in successive alcohol baths. Cells were imaged at 2 kV using a FEI-Quanta 250 SEM-FEG.

### SMAD assay using luciferase reporter gene

C2C12-A5 cells that were stably transfected with an expression construct (BRE-Luc) containing a BMP-responsive element fused to the firefly luciferase reporter gene (Logeart-Avramoglou et al., 2006), were generously gifted by D. Logeart-Avramoglou (Univ Paris Diderot). Cells were cultured and transfected under the same conditions as wild type C2C12 cells, and seeded in 96-well plates at 30,000 cells/cm^2^ in GM. After 24 h, cell lysis and luciferase measurements were carried out according to the manufacturer’s instructions (Bright-Glo™ luciferase assay system luminescence, Promega, France) as previously described (Crouzier et al., 2011a). Measurements were normalized to the DNA content of each sample as measured by the CyQUANT assay (C7026, Life Technology, France).

### Measurement of ALP activity in C2C12

At D3, cells seeded in 24-well plates were rinsed in PBS, lysed in deionized water and stored at −80°C. Lysates were then sonicated and centrifuged at 10,000 rpm for 5 min. To measure the ALP activity, 20 µl of the supernatants of lysates were added to 180 µl of a mixture (0.1 M 2-amino-2-methyl-l-propanol (Sigma-Alrich, St Quentin-Fallavier, France), 1 mM MgCI_2_, 9 mM p-nitrophenyl phosphate (pNPP) (Euromedex, Mundolsheim, France), pH 10) in 96-well plates. The ALP activity was assayed by measuring the absorbance at 405 nm using a Multiskan EX plate reader (Labsystem, Helsinki, Finland). Total protein amounts of each lysate were quantified using a BCA protein assay kit (Interchim, Montluçon, France). The activity was expressed as a percentage relative to the activity of the positive control.

### Immunoblotting and dot blot

Cells were lysed in Laemmli buffer (Sigma-Aldrich, France). Detection of proteins by Western blotting was done according to standard protocols. Extracellular matrix proteins were detected using dot blot. 1 µl of each lysate was deposited in triplicate onto a nitrocellulose membrane. Western blot and dot blot membranes were blocked at room temperature for 1 h in (10 mM Tris at pH 7.9, 0.15 M NaCl, 0.5% Tween 20, 5% w:w dry milk). The membranes were incubated with the primary antibodies (Supplementary Table S2) diluted in the blocking solution overnight at 4°C. After incubation with horseradish peroxidase secondary antibodies, detection of adsorbed antibodies was performed by ECL (Amersham Biosciences, France). Normalization was done by calculating an intensity ratio, taking actin as reference.

### SiRNA interference

Cells were transfected with siRNA against β1, β3, β5,M-cad, N-cad and cadherin-11 (ON-TARGET plus SMARTpool, Mouse, Thermo Scientific Dharmacon, France) (Supplementary Table S3). At the same time, a scrambled siRNA (All Stars negative Control siRNA, Qiagen) was used as a control. The transfection was done as previously described (Fourel et al., 2016). Briefly, cells were seeded at 50,000 cells per well in a 6-well plate and cultured in GM (2 ml per well). After 15 h and 39 h respectively, GM was replaced by GM without antibiotics before adding the pre-incubated transfection mix (for one well: 6 µl of lipofectamine RNAiMAX Reagent (Invitrogen), 610 µl of Opti-MEM medium (Gibco, France), and 1.44 µl of 50 µM siRNA). 24 h after the second transfection, cells were detached using trypsin-EDTA (Gibco, France) and seeded on TCPS or polyelectrolyte films.

### Data representation and statistical analysis

For box plots, the box shows 25, 50 and 75% percentiles, the square shows the mean value and the error bars correspond to the standard deviation. For scatter plots and bar plots, the mean values and the standard error of the mean (SEM) are represented. Experiments were performed at least in triplicate (biological replicates) with two wells per condition (technical replicate) in each experiment. Statistical tests were done using non-parametric Kruskal–Wallis ANOVA (*p < 0.05; **p < 0.01). To obtain normalized gene expression fold increase of the differentiation markers, first the mean gene expression values, previously normalized by the reference genes, of each experiment was calculated. Then, for each experiment, values were normalized by the value at time 0 (seeding of cells). Finally, the mean value and the SEM were calculated from the biological replicates. In the knockdown experiments, the relative values were calculated by first averaging all the mean values per well from all the experiments, then normalizing the values by the scrambled condition.

## Results

### Soluble and matrix-bound BMP-2 drive the muscle-osteoblast transdifferentiation

Here BMP-2 was either presented in solution for cells cultured on plastic (soluble BMP-2, sBMP-2) or presented to cells *via* a biomaterial in a matrix-bound manner (bBMP-2), the BMP-2 being physically trapped in a polyelectrolyte film made of hyaluronic acid and poly (L-lysine), as the “matrix” (Crouzier et al., 2011a). bBMP-2 on the film was used to mimic *in vitro* the BMP-2 presentation *in vivo,* where BMP-2 is bound in a non-covalent manner with the proteins and glycosaminoglycans of the extracellular matrix. When BMP-2 is presented *via* the polyelectrolyte film (Crouzier et al., 2009; Crouzier et al., 2011a; Fourel et al., 2016), it interacts with the BMP receptors at the basolateral side of the cells, where cells adhere (Crouzier et al., 2009; Crouzier et al., 2011a). Indeed, we already showed that C2C12 myoblasts begin the differentiation process to bone cells after their culture for 24 h in the presence of sBMP-2 or bBMP-2 (Crouzier et al., 2011a; Fourel et al., 2016). The presence of both sBMP-2 (Katagiri et al., 1994) and bBMP-2 (Crouzier et al., 2009; Crouzier et al., 2011a) inhibit the formation of myotubes and induce osteoblastic differentiation, as characterized by morphological changes, phosphorylation of SMAD and alkaline phosphatase activity.

To follow the muscle to osteoblast transdifferentiation process, we first analyzed the ability of sBMP-2 and bBMP-2 to inhibit muscle-specific transcription factors and to activate bone-specific transcription factors over the first 5 days of cell culture (Figures 1A–E). First, we verified that sBMP-2 induces effective transdifferentiation by following gene expression. After 1 day in DM without BMP-2, the expression of muscle-specific transcription factors mRNA such as MyoD and Myogenin, reached a plateau at 10 and 10,000 times higher, respectively, than before differentiation induction (named hereafter D1) (Figures 1B, C). Second, we imaged the presence of the transcription factors in the cells (Figures 1B′, C′). The increase of MyoD and Myogenin mRNA is correlated with an increase in their presence in the nucleus for 60% of cells for MyoD at D1 of differentiation and 35% of cells for Myogenin after D3 (Figures 1B′, C′), which was similar for sBMP-2 and bBMP-2. The percentage of positive cells for MyoD and Myogenin shows that both transcription factor translocate to the nucleus in the absence of BMP-2 (Figures 1B″, C″). In contrast, in the presence of sBMP-2, the mRNA specific to osterix and osteocalcin (Figures 1D, E), two hallmarks of bone differentiation (Katagiri et al., 1994; Celil et al., 2005), reached a plateau at ∼100 to 1,000 times higher value than their initial value at D-1 (Figures 1D, E). In addition, BMP-2 enhanced the translocation of osterix to the nucleus, which was particularly notable for bBMP-2 in comparison to sBMP-2 (Figures 1D′, D″). Finally, we assess whether sBMP-2 and bBMP-2 were able to induce matrix mineralization (Figures 1E′, E, E″). Similarly to sBMP-2, bBMP-2 is able to induce matrix mineralization as confirmed by Alizarin red staining at 3 weeks (Figure 1E′). Using phase contrast microscopy, we observed that cell morphology changed in response to BMP-2. In the absence of BMP-2, cells initially exhibit a fibroblast-like morphology and then form myotubes that are particularly visible at day 5 on TCPS and on films in the absence of BMP-2. In the presence of sBMP-2 and bBMP-2, cell shape changes from fibroblast-like to more a polygonal morphology at day 3 before bone nodule formation at day 5 in case of bBMP2 (Supplementary Figure S1). This polygonal morphology is consistent with the physiological cuboidal morphology of osteoblast cells (Roberts et al., 2011). The typical shape of calcium aggregates observed by scanning electron microscopy (SEM) confirmed the mineralization triggered by the presence of sBMP-2 and bBMP-2 (Figure 1E″). To note, the presence of calcium and phosphate in these aggregates was confirmed by energy dispersive X-ray analyses (data not shown).

Together our results show that both sBMP-2 and bBMP-2 are able to induce cell differentiation to osteoblasts, cells expressing muscle markers in the absence of BMP-2 and osteoblast markers in the presence of sBMP-2 and bBMP-2. The bioactivity bBMP-2 was also sufficiently stable to induce longer-term osteogenesis and mineralization. Thus, like sBMP-2, bBMP-2 inhibits the myogenic differentiation of skeletal progenitors to osteoblast cells.

### sBMP-2 and bBMP-2 induce the expression, secretion and remodeling of an osteoblast-specific extracellular matrix

**FIGURE 2:**
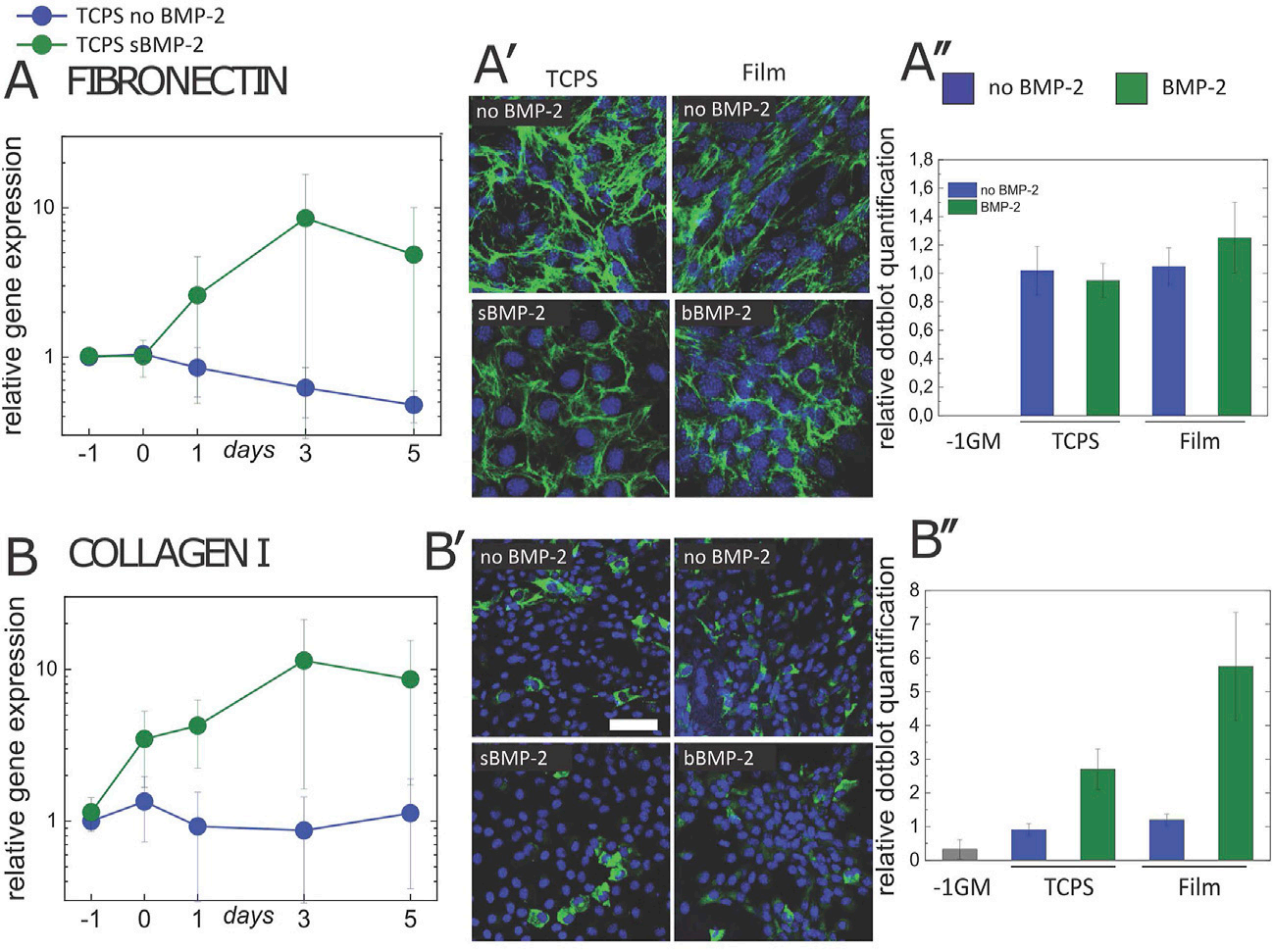
bBMP-2 and sBMP-2 induce the gene expression, secretion and remodeling of fibronectin and collagen I in C2C12 cells. **(A,B)** Gene expression of FN and COLL1 were followed up to D5 in the presence (green) or absence (blue) of sBMP-2 on TCPS. At D5 for cells grown on films with bBMP-2 or on TCPS with sBMP-2 **(A′)** FN and **(B′)** COLL1 were stained using immunofluorescence and quantified by dot blot **(A'',B''** respectively**)**, by taking actin as a control. Scale bars are 150 µm. Data are mean ± SEM of three independent experiments.

Next, the production of osteoblast-specific extracellular matrix was assessed by qPCR, immuno-fluorescence staining and Western Blot for cells cultured on TCPS in the presence of sBMP-2 or on films with bBMP-2. Fibronectin is a ubiquitous protein present in a large number of tissues including bone and muscle (Vogel, 2018). The gene expression of fibronectin (FN) and collagen I (COLL1), two major proteins of bone tissue (Moursi et al., 1997; Brunner et al., 2011), increased on TCPS in the presence of sBMP-2 (Figure 2A). Fibronectin organization around the cells was modified by the presence of BMP-2 (Figure 2A′): in the absence of BMP-2, cells stretched out FN in long, thin fibers, following cell alignment. In the presence of BMP-2, FN appears as short, thin fibers between cells, following the polygonal shape of the cells and delineating the cell boundary. At the protein level, fibronectin expression was not significantly different in response to sBMP-2 or bBMP-2 (Figure 2A″).

The expression of collagen I at the gene level (COLL1) notably increased within 2 days in the presence sBMP-2 (Figure 2B). The plateau was about 10-fold higher than the initial value at D1.

Immunofluorescence staining of collagen at the protein level did not reveal a difference between conditions, the expression of collagen in C2C12 cells being mostly localized inside the cells (Figure 2B′). However, quantification of the expression of collagen protein by dot blot revealed a 3-fold increase in collagen production in the presence of BMP-2 (Figure 2B″).

The absence of collagen fibrils outside the cells in our experimental procedures may be due to the lack of ascorbic acid (vitamin C). Indeed, in absence of vitamin C, a co-factor of the prolyl-4 hydroxylase, collagen molecules are thermodynamically unstable, which prevents their assembly into fibrils in the extracellular space (Salamito et al., 2022). Together, these results indicate that, in addition to the production of osteocalcin (Figure 1E), the presence of both soluble and matrix-bound BMP-2 induces changes in the two major osteogenic extracellular matrix: increase in collagen one production and reorganization of fibronectin.

### BMP-2 leads to a tissue-specific switch in the expression of the adhesion receptors integrins and cadherins

**FIGURE 3:**
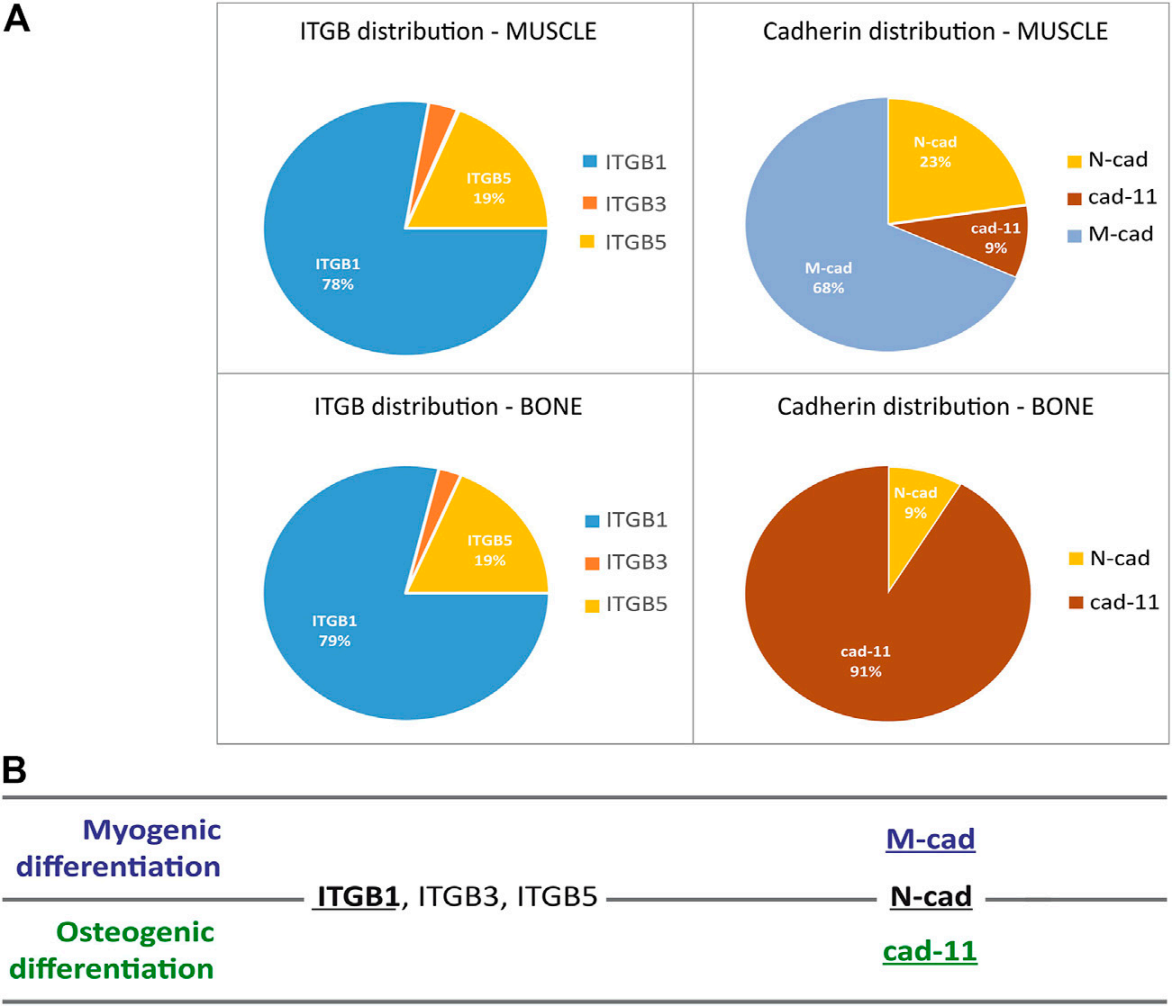
Identification using the ENCODE database of muscle and osteoblast-specific integrins and cadherins. **(A)** Pie chart of the percentage of expression of ITGB1, three and five chains **(A)** and M-, N- and cadherin and cadherin-11 in muscle and osteoblast cells. Data were obtained by analyzing RNA sequencing data made for the ENCODE public research project i.e., charts illustrate the predominance of certain adhesion receptors in each cell type. **(B)** Summary table highlighting the adhesion receptor repertoire for myogenic and osteogenic differentiation and the receptors that are important for both tissues. The underlined adhesion receptors are the most studied in the literature in the context of myogenic and bone differentiation.

In view of the large number of adhesion receptors, an *in silico* screening was performed using RNA sequencing data from UCSC genome browser (ENCODE data base). This website offers access to the genome sequencing of many species as well as RNA sequencing. This database allowed us to identify adhesion receptors expressed in myotubes and in osteoblasts and to express their relative abundance by quantifying the percentage. First, the distribution of adhesion receptors in each cell type, muscle and osteoblast, was analyzed for cadherins and integrins β chains (genes named hereafter ITGB) (Figure 3A), and for α chain integrins (ITGA, Supplementary Figure S2) knowing that the functional unit of integrins is a heterodimer made of α and β chain and multiple heterodimers bind the same ligand (Humphries et al., 2006) The ITGB pie chart was similar for muscle and osteoblast cells, ITGB1 being the most expressed (78%–79%) followed by ITGB5 (19%) and ITGB3. Expressions of other ITGBs, which were below 1%, were not represented in this figure. The expression pattern of ITGAs allowed us to better discriminate cell specificity, i.e., muscle *versus* osteoblast cells (Supplementary Figure S2). In decreasing order, muscle cells mostly expressed ITGA5 (28%), ITGA7 (27%) and ITGA3 (20%) followed by ITGA6 (10%) and ITGAV (8%), while osteoblast cells expressed ITGA11 (43%), ITGAV (25%) and ITGA5 (24%). M-cad (68%) and N-cad (23%) were the most expressed cadherins in muscle cells (Charrasse et al., 2003), while cad-11 was the most expressed cadherin in osteoblast cells at 91% (Alimperti et al., 2014). To note, N-cad was equally expressed in osteoblast and muscle cells. Panel 3B summarizes the role of integrins and cadherins in myogenic and osteogenic differentiation. This data suggest that there is a clear shift of cadherin receptor upon BMP-2 stimulation while all three β chain integrins are present, their role being indistinguishable at first sight by solely looking at their gene expression.

**FIGURE 4:**
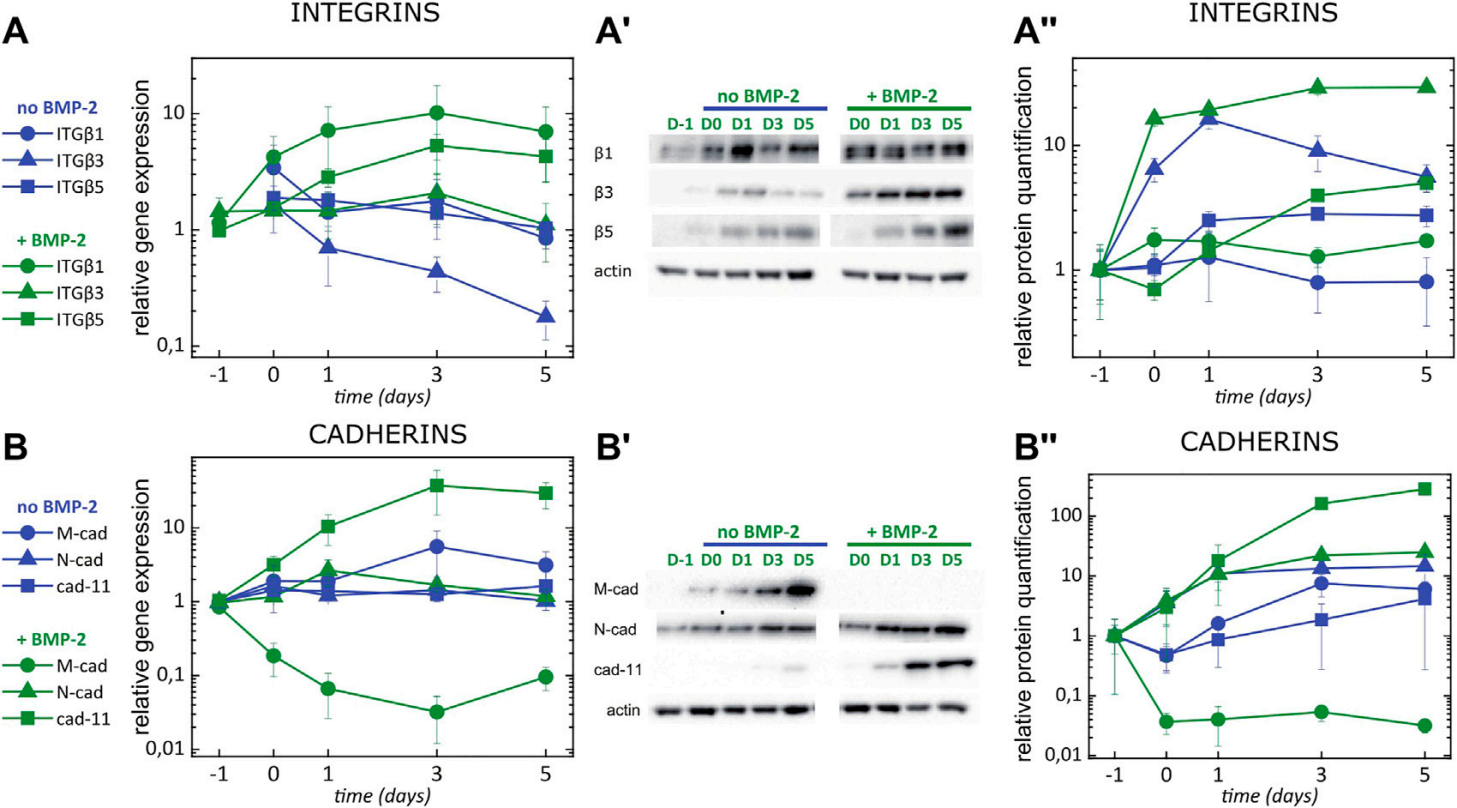
bBMP-2 and sBMP-2 induce the expression switch towards specific integrins and cadherin 11 in C2C12 cells. Kinetics of gene expression of **(A)** ITGB chains **(B)** cadherins quantified by RT-qPCR for cells cultured on TCPS without (blue) or with (green) sBMP-2 in solution. **(A′,B′)** Western blots and **(A'',B'')** corresponding analysis of kinetics of protein expression. Actin was taken as control for intensity normalization. Data are mean ± SD of three independent experiments.

To identify which adhesion receptors were important in the muscle to osteoblast transdifferentiation, we quantified the kinetics of gene expression by qPCR and the protein expression by western blot as a function of time, over 6 days (Figure 4). In addition to the β1 integrin that was already known as a major integrin in bone formation, β3 and β5 integrins appear as important integrins during the muscle to bone transdifferentiation in response to BMP-2 (Figures 4A′, A″). Based on the protein expression, β5 integrin appears to be expressed at later times than β3, whose expression appears as soon as few hours of BMP-2 stimulation.

The relative expression of α chain integrins determines ligand specificity in particular for FN (Supplementary Figure S3) and for COLL1 (Supplementary Figure S4) discriminating osteoblast and muscle differentiation. α5 (complexed with β1 subunit) and αV (complexed with β1 or β3 subunit) are known to be FN specific integrins (Moursi et al., 1997) while α1, α2 and α11 (complexed with β1 subunit) are known to be COLL1 specific (Pozzi et al., 1998; Xiao et al., 1998). The α5 and αV chains involved in FN recognition were expressed, with an increase in αV expression in response to sBMP-2, as quantified by qPCR and western blot (Supplementary Figures S3 A, A′, A″) whereas the expression of ITGA1, ITGA2 and ITGA11 notably increased in the presence of sBMP-2 (Supplementary Figure S4).

Finally, cadherin expression was also affected by the presence of BMP-2 (Figures 4B, B′,B″), as anticipated from the switch in cadherins revealed by Encode analysis (Figure 3). At the gene level, cad-11 notably increased in the presence of sBMP-2 while M-cad was downregulated (Figure 4B). Both cad11 and Ncad increased at the protein level, indicating osteoblast-specific differentiation (Figure 4B′, B″). To note, the increase of Ncad expression in response to BMP-2 was early, after few hours, while cad11 expression increased strongly at the later time points, after day 3.

Overall our results demonstrate a switch of the integrin and cadherin repertoire upon BMP-2 stimulation, thus highlighting a potential role of integrin and cadherin receptors in osteoblast differentiation. β3 and β5 emerge as osteoblast-specific integrins associated with the extracellular matrix transition during the transdifferentiation, while Cad-11 and N-cad emerge as the key cadherins. Both N-cad and β3 integrins appear to be involved at early times.

### Biomimetic films with matrix-bound BMP-2 enable to reveal the BMP-2 mediated transcriptional activity

**FIGURE 5:**
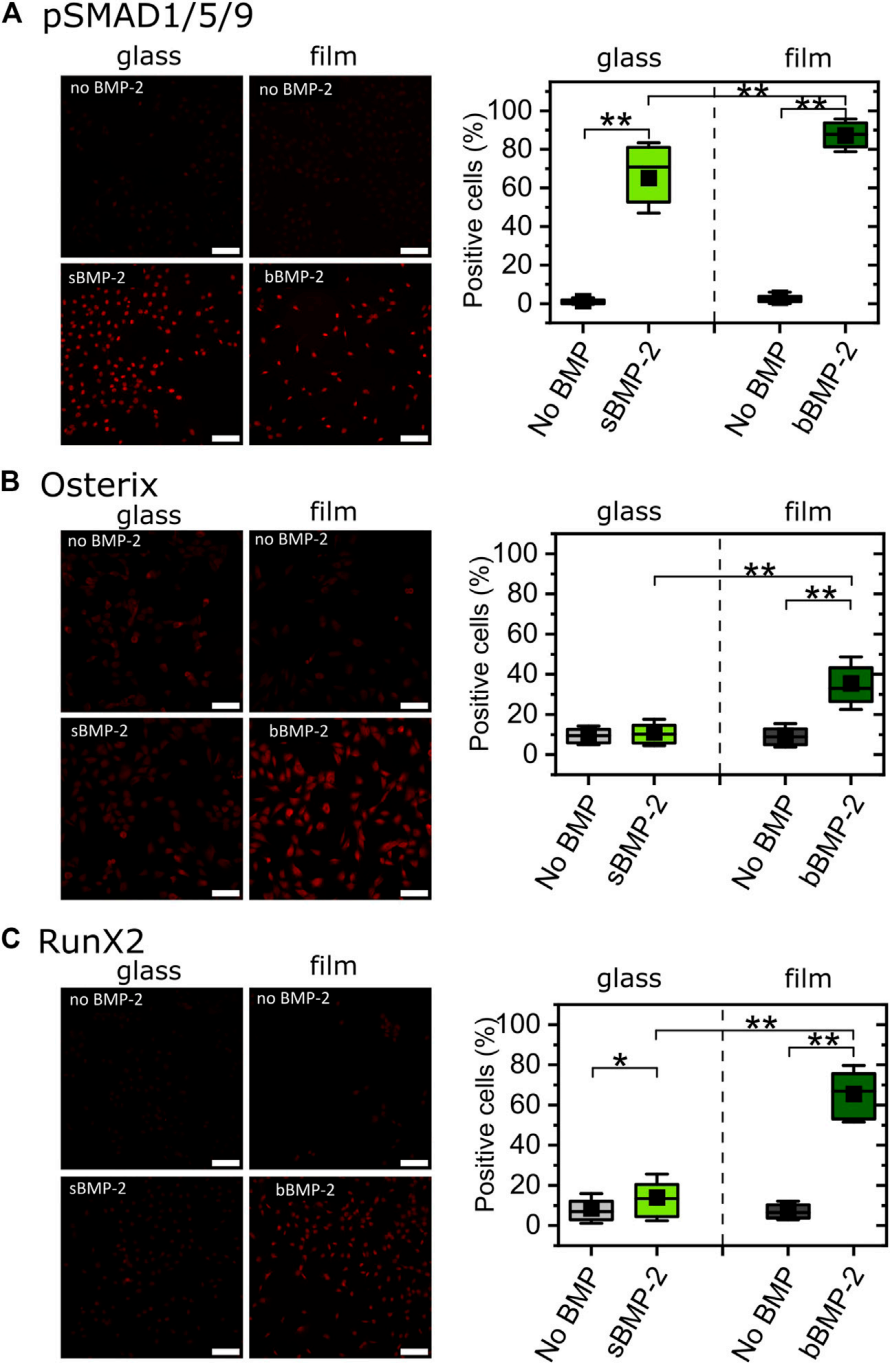
Quantification of the nuclear localization of selected transcription factors in response to soluble and matrix-bound BMP-2. Selected transcription factors that are representative of bone differentiation, i.e., **(A)** pSMAD1/5/9, **(B)** osterix, and **(C)** RunX2, were quantified for C2C12 myoblasts grown on glass in the presence or absence of sBMP-2 and for cells cultured on biomimetic films with or without matrix-bound BMP-2. Representative immuno-fluorescence images of cells cultured in the different conditions are shown. Scale bar of fluorescent images is 100 µm. The corresponding quantifications of the nuclear location of the transcription factors based on the immuno-fluorescence are given for cells in the presence of sBMP-2, bBMP-2 and in the absence of BMP-2. The % of positive cells is given for each condition. Data are represented as box plots for three independent experiments.

Cell differentiation is characterized by specific transcription factors. We investigated the nuclear localization of three transcription factors known to be important in osteoblast differentiation, pSMAD, osterix (da Silva Sasso et al., 2021), RunX2 (Komori, 2022) (Figure 5). We quantified their expression for cells grown on TCPS in the presence of sBMP-2 and for cells grown on biomaterials with bBMP-2 (Figures 5A–C). To this end, the transcriptions factors were immuno-stained and their nuclear localization was quantified using automated image analysis. Representative images of each experimental condition are shown. Quantitatively, we compared the percentage of positive cells in the absence of BMP-2 and in the presence of sBMP-2 or bBMP-2. For pSMAD1,5,9, the % of positive cells was above 75% for both sBMP-2 and bBMP-2 (Figure 5A). For both osterix (Figure 5B) and RunX2 (Figure 5C), a significant increase in the % of positive cells was noted for cells with bBMP-2, in comparison to sBMP-2. Thus, the biomimetic films with matrix-bound BMP-2 enable to potentiate the BMP-2 mediated cell response, in comparison to sBMP-2, with a significant increase in the nuclear localization of Osterix and RunX2.

### BMP-2 orchestrates an integrin-cadherin cross-talk to drive osteoblast differentiation

We next investigated the role of integrins and cadherins in the BMP-2 induced cell response. We chose to work solely with biomaterials presenting bBMP-2 in order to be in the experimental conditions with the highest expression of the transcription factors, as bBMP-2 effects were stronger than those of sBMP-2 (Figure 5).

**FIGURE 6:**
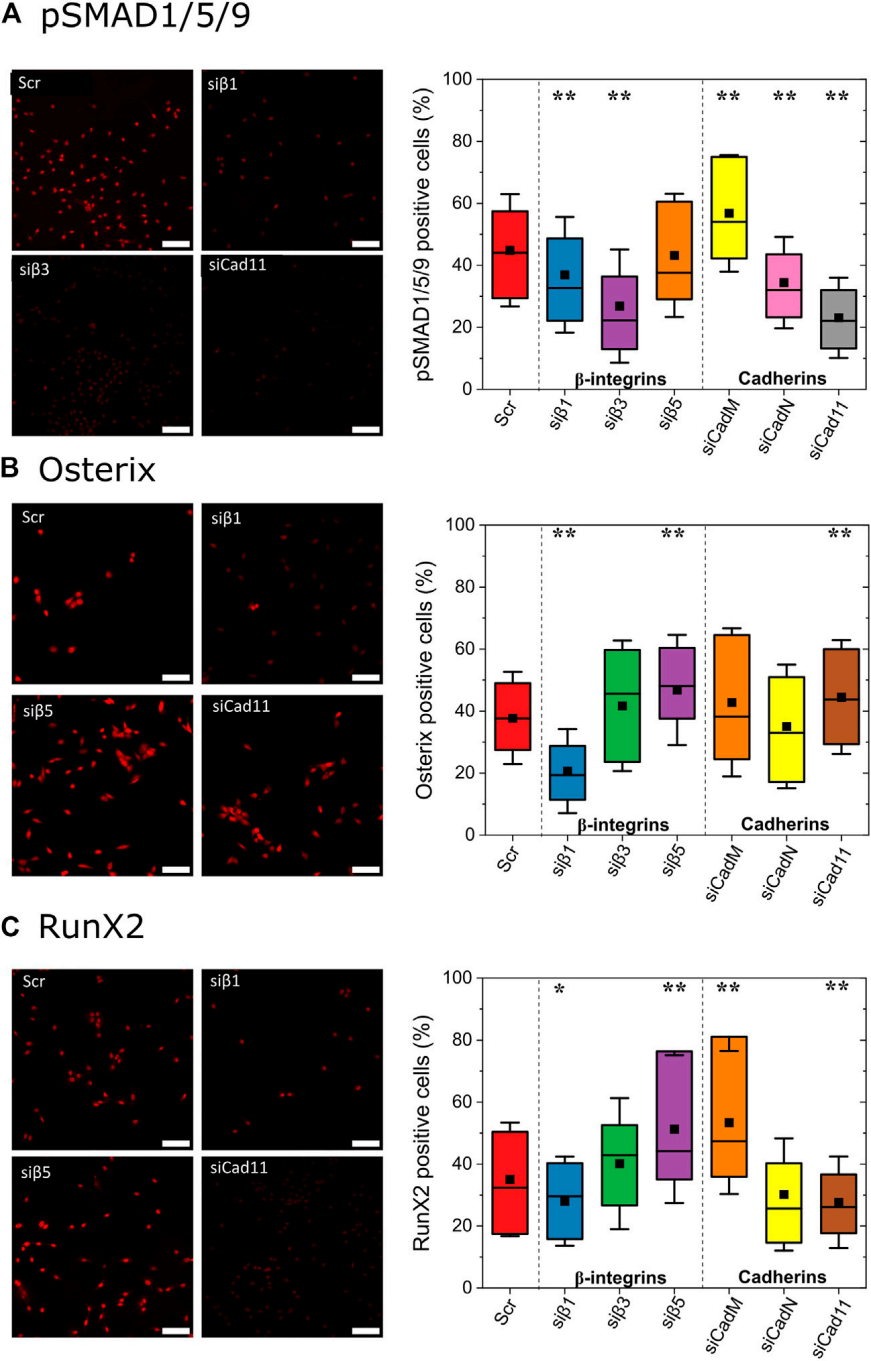
Role of β chain integrins and cadherins on the nuclear localization of transcription factors involved in osteoblast differentiation. Using RNA interference, silencing of the major integrins (β1, 3, 5 subunits) and cadherins (M-cad, N-cad, cad11) was done to assess their individual role on the nuclear localization of **(A)** pSMAD1,5,9, **(B)** Osterix and **(C)** Runx2, for cells cultured on films with bBMP-2. Representative images are shown for selected conditions that have the strongest effects on the nuclear localization of the transcription factor. Scale bar of fluorescent images is 100 µm. Data are represented as box plots for three independent experiments. Statistical tests were done using non-parametric Kruskal–Wallis ANOVA (*p < 0.05; **p < 0.01).

To investigate the roles of integrins and cadherins in the nuclear localization of the transcription factors, we used silencing RNA to knockdown integrin β chains and cadherin adhesion receptors (Figure 6). The efficiency of the receptor silencing was confirmed by qPCR (Supplementary Figure S5). Regarding pSMAD1,5,9 (Figure 6A), the deletion of β3 integrin or cadherin11 led to statistically significant decrease with ∼25% signal extinction as compared to the control, while the deletion of β1 integrin or N-cadherin generated a smaller decrease of ∼15%. For Osterix (Figure 6B), the deletion of β1 integrin significantly decreased the nuclear signal by 25% and that of N-cadherin also decreased the nuclear signal. In contrast, deletion of β5 integrin or of Cadherin11 led to an increased nuclear location. For RunX2 (Figure 6C), deleting β1 and cad11 decreased its nuclear localization, while deleting β5 integrin or M-Cadherin increased its nuclear localization by ∼15%.

Globally, β3 integrin controlled more strongly the pSMAD 1,5,9, nuclear location; β1 integrin controlled osterix and RunX2 nuclear locations; and β5 integrin played an opposite role by preventing the nuclear location of Osterix and RunX2.

Regarding cadherins, Cadherin 11 had the strongest role in inducing nuclear location of pSMAD and RunX2, while it was a negative regulator of osterix. M-Cadherin had globally an opposite effect. N-Cadherin played a significant role solely for pSMAD1,5,9. It is interesting to note that β3 integrin and N-Cadherin played an important role solely for pSMAD nuclear location.

**FIGURE 7:**
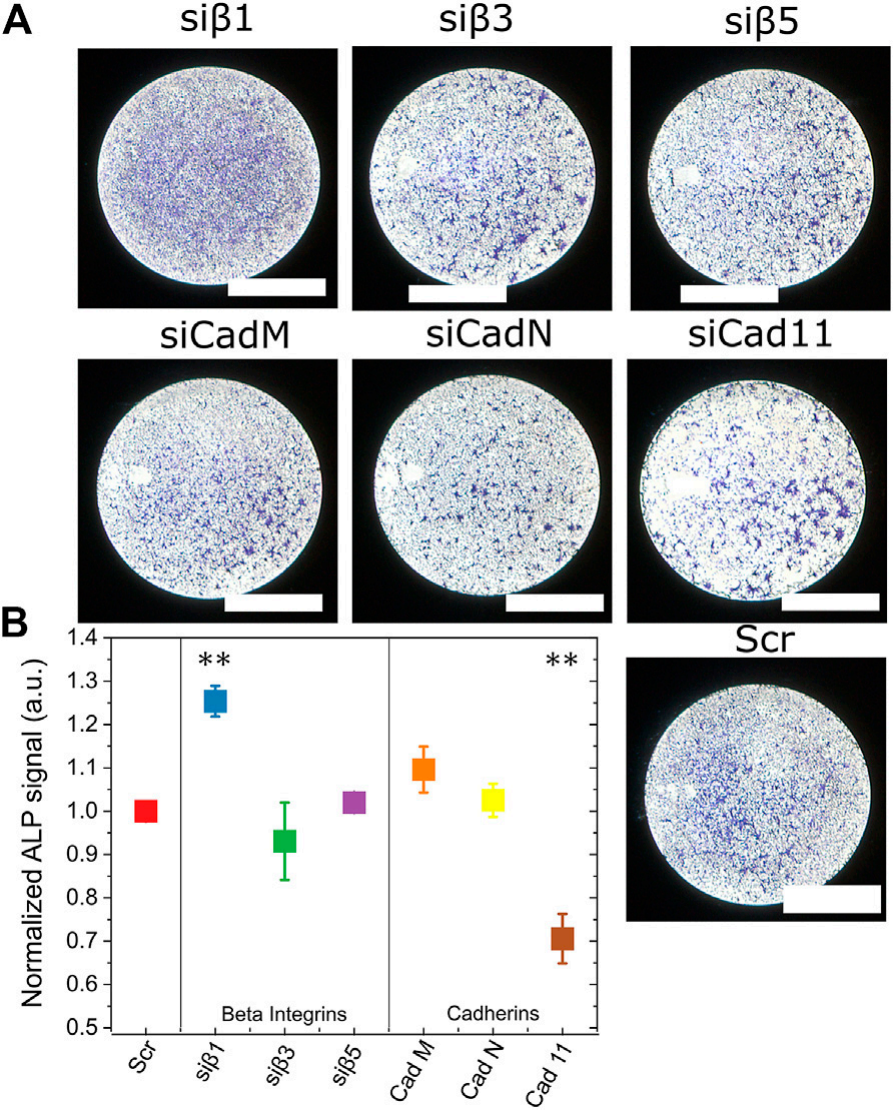
Role of β chain integrins and cadherins in ALP expression. ALP expression was quantified after silencing of the three major β chain integrins and cadherins (same conditions as in Figure 6) **(A)** Representative images of a single well of a 96-well plate are shown for each silencing condition. **(B)** Quantification of alkaline phosphatase activity (ALP) after 4 days of C2C12 cell culture (24H in GM and 72 H in DM) on films with matrix-bound BMP-2, after RNA silencing of each β chain integrin and cadherin receptor. In each experiment, data were normalized to the scramble condition. Data are mean ± SEM of three independent experiments. Statistical tests were done using non-parametric Kruskal–Wallis ANOVA (*p < 0.05; **p < 0.01).

Next, we investigated the role of integrins and cadherins on ALP activity, which was quantified after 3 days of culture on biomaterials presenting bBMP-2 (Figure 7). Representative images of single wells of a 96-well cell culture microplate are shown for each of the silencing conditions (Figure 7A). Quantification of the ALP signal inside each well revealed that only β1 integrin and Cadherin 11 strongly impacted ALP production (Figure 7B). However, they have opposite roles: β1 integrin was a negative regulator of ALP activity, while Cadherin 11 was a positive regulator of ALP. The other adhesive receptors did not have a significant impact on ALP activity.

To conclude, β3 integrin and N-Cadherin might couple to activate pSMAD, while β1 integrin and cadherin11 may couple to activate osterix and ALP.

### Fibronectin organization in response to bBMP-2

**FIGURE 8:**
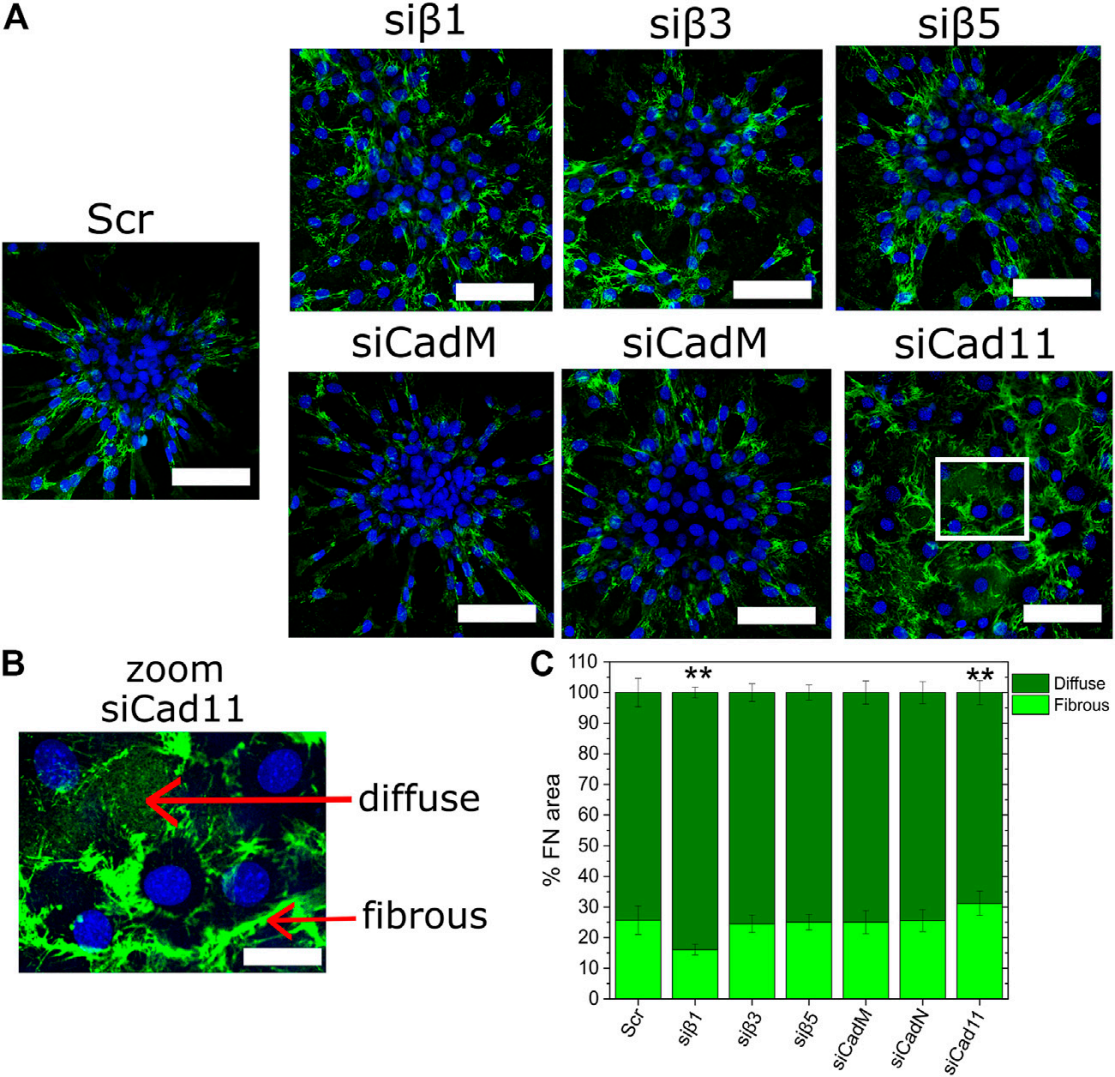
BMP-2-induced fibronectin remodeling depends on ITGB1 and cad-11. **(A)** Microscopic observations of fibronectin network after silencing using RNA of ITGB1, 3, 5 and M-cad, N-cad, cad-11 (same as in Figures 6, 7). Fibronectin (green) and actin (blue) were stained by immunofluorescence after 4 days (24 h in GM and 3 days in DM) of C2C12 cell cultured on films with matrix-bound BMP-2. **(A)** Representative image of each of the silencing condition. Scale bar are 50 µm. **(B)** Zoom on two conditions: silencing of β1 integrins and cadherin 11. Scale bar is 20 µm. **(C)** Quantification of the areal fraction of diffuse and fibrous fibronectin given: the total is set to 100 and the fraction represents the importance of each population. Data are mean ± SD of three independent experiments. Statistical tests were done using non-parametric Kruskal–Wallis ANOVA (*p < 0.05; **p < 0.01).

After having shown an integrin and cadherin switch upon BMP2 stimulation, we next asked whether the organization of osteoblast-like extracellular matrix might result from a BMP-2-induced coupling between integrins and cadherins. To address this question, we analyzed fibronectin deposition and remodeling in response to BMP-2 with SiRNA directed against cadherins and ITGBs (Figure 8). As bBMP-2 effects were stronger than those of sBMP-2 (Figure 5), with a very high response to bBMP-2 in comparison to the condition without BMP-2, these experiments were done on films with bBMP-2 in order to more easily capture the possible effects of silencing RNA. In the control scramble condition, cells tend to gather forming bone nodules encircled by FN (Figure 8A). In contrast, the deletion of β1 integrin and cadherin11 led to a disorganized FN deposition (Figure 8A) while the deletion of other integrins and cadherins did not strongly impact bone nodule formation and FN organization. Long and aggregated fibers were observed after silencing β1 integrin and cadherin 11, compared to thin, short and numerous fibers in the control condition (Figure 8B). Quantification of FN organization in diffuse or in a fibrous morphology (Figure 8C) showed that there were significant differences for β1 integrin and cadherin 11, with more diffuse FN upon deletion of β1 integrin and more fibrous FN upon deletion of Cadherin11. In addition to osteoblast cell reprogramming of Figures 6, 7, these results highlighted a key role of both β1 integrin and cadherin 11 in driving extracellular matrix organization in response to matrix-bound BMP2 presented by the biomimetic films.

## Discussion

Upon BMP-2 stimulation, the osteoblastic lineage commitment in C2C12 myoblasts is associated with a microenvironmental change that occurs over several days (Ozeki et al., 2007). This change implies adapted cell interactions with the extracellular matrix and neighboring cells. Cell interactions with extracellular matrix and cell-cell interactions are mediated by integrins and cadherins, respectively, and are critical for osteoblast tissue morphogenesis and architecture (Marie et al., 2014). Our findings highlight the switch of integrin and cadherin expression during the muscle to osteoblast transdifferentiation in response to stimulation by soluble or matrix-bound BMP-2. While C2C12 muscle cells express M-cad and Laminin-specific integrins, the BMP-2-induced transdifferentiation to osteoblast cells is associated with an increase in the expression of cad11 and fibronectin (Moursi et al., 1997; Globus et al., 1998) and collagen-specific integrins, in agreement with the literature (Lai et al., 2006; Di Benedetto et al., 2010; Greenbaum et al., 2012) (Moursi et al., 1997; Globus et al., 1998; Zimmerman et al., 2000; Brunner et al., 2011). Our study reveals how integrins and cadherins can work in concert to drive the osteogenic differentiation and to control osteoblast cell microenvironment. Indeed, our results show that different sets of integrins and cadherins act at different times of muscle-osteogenic transdifferentiation.

### Interplay between N-cadherin/β3 integrin and Cadherin11/β1 integrin to regulate transcriptional activities

Our previous work demonstrated that there is a crosstalk between BMP receptors and β3 integrin in the early steps of myoblast to osteoblast transdifferentiation (Fourel et al., 2016). Indeed, we showed that β3 integrin plays a role in SMAD signaling (Fourel et al., 2016). We proposed a model wherein β3 integrin is a key element that acts at early steps in a multistep process by controlling both the phosphorylation of SMAD1 by BMPR and the stability of pSMAD1 through the repression of GSK3 activity. Here, we showed the early pSMAD1,5,9 is regulated both by β3 integrin and by N-cadherin (Figure 6). Our results are consistent with the role of β3 integrin in early osteogenic differentiation (Yuh et al., 2020), which is associated with tensile loading (Peng et al., 2021).

At later stages of the differentiation, β1 integrin is involved in the control of other transcription factors like Osterix and RunX2 and ALP expression, upon BMP-2 stimulation. This is in agreement with the known role of β1 integrin in osteoblast differentiation (Celil et al., 2005; Lai et al., 2006). However, we noted that β1 integrin is a negative regulator of ALP expression. Cadherin 11 is strongly involved in the positive regulation of pSMAD1,5,9 RunX2 and ALP, and as a negative regulator of osterix. Interestingly, β5 integrin appeared as a negative regulator of Osterix and RunX2 (Figures 6, 7). β5 integrin may control osteogenesis by regulating Wnt/β-catenin signaling through its interaction with the protein half LIM domains protein 2 (FHL-2) (Martin et al., 2002; Lai et al., 2006; Hamidouche et al., 2009).

Controlling the activity of GSK3 (Fourel et al., 2016) might be an intermediate step for adhesive receptors to control osteogenic genes. Indeed, GSK3 inhibition is central to control both the intensity and the duration of SMAD and non-SMAD signals. Of note β5 integrin and GSK3 have been both identified as osteosarcoma markers (Le Guellec et al., 2013). In addition, N-cadherin is involved in GSK3 and β-catenin phosphorylation through Akt activation (Zhang et al., 2013) and mediates osteogenesis by regulating Osterix through PI3K signaling and GSK3 (Guntur et al., 2012). In another study, mechanical strain was found to regulate osteoblast proliferation through integrin-mediated ERK activation, with β1 and β5 integrins having opposite effects. (Yan et al., 2012).

### Cooperation between cadherin11 and β1 integrin in controlling extracellular matrix and tissue mechanics

Mechanotransduction, which is the transduction of mechanical forces to biochemical signals, is an important mechanism regulating both cellular and matrix mechanics to control osteoblast maintenance and regeneration (Shih et al., 2011). In line with this, our most intriguing result was the reorganization of FN during transdifferentiation of C2C12 cells (Figure 2) and the role of β1 integrin/cadherin11 in this process (Figure 8). Our experiments did not show that the global amount of FN increased in the presence of BMP-2. This is in contrast to previous studies of other cell types and with different experimental conditions (Perez et al., 2011; Fourel et al., 2016). Instead, our results showed differences in FN organization. While C2C12 cells elongated FN fibrils in the absence of BMP-2, FN localized at cell-cell contacts with short fibrils, in the presence of bBMP-2 (Figure 2 and Figure 8A) with is associated with the ability to form bone nodules characterized by cell gathering (Figure 8A). Deletion of β1 integrin or Cadherin11 disrupt the ability of cells to form bone nodules (Figure 8A) through different impact of β1 integrin and Cadherin11 on FN organization. Many data have shown that β1 integrin plays an important role in osteoblast differentiation and function (Moursi et al., 1997; Xiao et al., 1998; Wang and Kirsch, 2006; Hamidouche et al., 2009; Brunner et al., 2011). Mice expressing a dominant-negative β1 integrin subunit in mature osteoblasts show reduced bone mass and defective bone formation (Moursi et al., 1997). The control of β1 integrin activation has been shown to be crucial in controlling matrix assembly and osteoblast differentiation (Brunner et al., 2011). Consistent with this, a study showed that Wnt-induced secreted protein-1 (WISP-1/CCN4) promotes MSC osteogenic differentiation *in vitro* by binding to integrin α5β1 and enhancing the anabolic effect of BMP-2 (Ono et al., 2011). WISP-1/CCN4 is a member of the CCN family of proteins, which are secreted extracellular matrix-associated proteins, that is highly expressed in skeletal tissues. Our results suggest a mechanical role of β1 integrin and cadherin11. Even though substrate stiffness and tethering is mostly known to affect focal adhesions (Levental et al., 2009; Yu et al., 2010b; Trappmann et al., 2012; Wen et al., 2014), increasing evidence suggests that it may also affect cadherin-mediated intercellular adhesion (Ladoux et al., 2010; Smutny and Yap, 2010). It has been already shown that cadherin11 and β1 integrin regulate both the contractile pathway through ROCK signaling to control extracellular matrix mechanics (Clark et al., 2005; Brunner et al., 2011; Faurobert et al., 2013; Alimperti et al., 2014). The question is whether cooperation between β1 and cadherin11 might affect intercellular stress or the strength of adhesion to the extracellular matrix as function of bone differentiation stages. Previous studies have shown quantitatively that cells exert different traction forces on pillars covered by fibronectin depending on the type of cadherins (Jasaitis et al., 2012), and each type of cadherin might be associated with specific intercellular adhesion strengths (Chu et al., 2004) (Lai et al., 2006). It has also been proposed that cadherin 11 regulates the cell-cell tension necessary for calcified nodule formation by valvular myofibroblasts (Hutcheson et al., 2013). How cadherin11 and β1 integrin can work together at the molecular scale is still not understood. Their unexpected localization may provide a part of the answer. Surprisingly cadherin 11 has been localized at cell-extracellular matrix contacts in focal adhesions (Langhe et al., 2016) and fluorescence cross-correlation spectroscopy has revealed the presence of the inactive form of α5β1 integrin at cell-cell contacts, which was under the control of N-cadherin in a zebrafish model (Julich et al., 2015). Altogether, these data suggest that BMP-2 might tune interplay between N-cadherin/Cadherin11 and integrin-dependent signals to control cell fate by regulating the strength of adhesion to the extracellular matrix, extracellular matrix remodeling and extracellular matrix mechanics.

## Data Availability

The original contributions presented in the study are included in the article/Supplementary Materials, further inquiries can be directed to the corresponding authors.
