# Supplementary material for: Interplay between integrins and cadherins to control bone differentiation upon BMP-2 stimulation
Source: Front Cell Dev Biol. 2023 Jan 4;10:1027334. doi: 10.3389/fcell.2022.1027334 (PMC9846056; doi:10.3389/fcell.2022.1027334)

Supplementary file Valat et al., V251122

Table SI 1. Primer sequences used in mouse C2C12 skeletal myoblasts.

Mouse Gene	Forward sequence (5'-3')	Reverse sequence (5'-3')
MYO D	ACTGCTCTGATGGCATGATGG	CACTGTAGTAGGCGGTGTCG
MYOGENIN	CAACCAGGAGGAGCGCGATCT	AGGCGCTGTGGGAGTTGCATTCACT
OSTERIX	TGCCTGACTCCTTGGGACC	CTTTGTGCCTCCTTTCCCCA
OSTEOCALCIN	CCGGGAGCAGTGTGAGCTTA	TAGATGCGTTTGTAGGCGGTC
FIBRONECTIN	GTGTTCAGCTCACTGGCTACC	TAGTGGCCACCATGAGTCCT
COLLAGEN 1	TGTGTGCGATGACGTGCAAT	GGGTCCCTCGACTCCTACA
ITGA5	CTTCTCCGTGGAGTTTTACCG	GCTGTCAAATTGAATGGTGGTG
ITGA11	AGCCTTTGGCCAGGATTCAC	CCATTGGTTTCCATTGGGGC
ITGAV	CCGTGGACTTCTTCGAGCC	CTGTTGAATCAAACTCAATGGGC
ITGβ1	CGGACGCTGCGAAAAGATGA	CACATCGTGCAGAAGTAGGC
ITGβ3	CCACACGAGGCGTGAACTC	CTTCAGGTTACATCGGGGTGA
ITGβ5	CCCGTTATGAAATGGCCTCA	GCCTAGCTAGCGTGAGCAAA
M-Cad	CCAGATTAACGTGAGCCATGC	GACGTTCAGCGTTTGCTCTC
N-Cad	CATCACATACGTCCCAGGCT	GCTGATAGCCCGGTTTCACT
Cad-11	CATCCTGAATGCCGGTCTGA	CAGCACAACGATGACCAGAAG
EF1	CCGTCAGAACGCAGGTGTTG	GTTCGCTTGTCGATTCCACC
PPIA	GTCTCCTTCGAGCTGTTTGC	GCGTGTAAAGTCACCACCCT
GUSB	CGGGACTTTATTGGCTGGGT	CCATTCACCCACACAACTGC

Table SI 2. References of antibodies.

Protein Target	Reference
MYO D	BD 554130
MYOGENIN	Santa Cruz sc-576
OSTERIX	Santa Cruz sc-22536-R
FIBRONECTIN	Sigma F3648
COLLAGEN I	Southern Biotec 1310-01
ITGA5	Millipore 1921
ITGAV	BD 611012
ITGB1	BD 610467
ITGB3	Emfret M030-0
ITGB5	Santa Cruz sc-14010
M-Cad	Nanotools 0106-100/MCAD-12G4
N-Cad	BD 610921
Cad-11	Cell signaling 13577

Table SI 3. Gene target siRNA sequences used for transfection.

Gene Target	Reference DHARMACON	siRNA target sequence (5' to 3')
β1 integrin	L-040783-01-0005, ON-TARGETplus Mouse Itgb1 (16412) - SMARTpool	UGCCAAAUCUUGCGGAGAA<br>UUACAAGAGUGCCGUGACA<br>GUGAAGACAUGGACGCUUA<br>CAAUGAAGCUAUCGUGCAU
β3 integrin	L-040746-01-0005, ON-TARGETplus Mouse Itgb3 (16416) – SMARTpool	AAACAGAGCGUGUCCCGUA<br>AAACACGUGCUGACGCUAA<br>GAGCAGUCUUUCACUAUCA<br>GUGAAAGAGCUGACGGAUA
β5 integrin	L-042453-01-0005, ON-TARGETplus Mouse Itgb5 (16419) - SMARTpool	CCGCUUAGGUUUCGGGUCU<br>GCUAGGCACGCACGGAUAA<br>AGAAGAUCGGAUGGCGAAA<br>ACUGCUAAGGACUGCGUUA
Cad-M	L-044301-01-0005, ON-TARGETplus Mouse Cdh15 (12555) - SMARTpool	CCAGGAUGCAUACGACAUA<br>UCGACGAGCACACGGGAGA<br>GAGAGAAGACGGACCGCUU<br>GAGCAAACGCUGAACGUCA
Cad-N	L-040206-00-0005, ON-TARGETplus Mouse Cdh2 (12558)- SMARTpool	GAUCAAAGCCUGGGACGUA<br>GUGCAACAGUAUACGUUAA<br>CAGAGAAUCGCCAAAUGUA<br>GGACAGGAACACUGCAAAU
Cad-11	L-053105-00-0005, ON-TARGETplus Mouse Cdh11 (12552) - SMARTpool	GCUUAUAGCUUGAAGAUAG<br>AGAUAACACUGCAGGAGUA<br>GGUCAUCGUUGUGCUGUUU<br>CAAUUGAUCGUCAUACUGA
Scrambled	D-001810-01-05) ON-TARGETplus Non-targeting	UGGUUUACAUGUCGACUAA

FIGURE SI1. Representative images of C2C12 myoblasts grown in the different culture conditions for up to five days. Cells were grown on tissue culture polystyrene in the absence of BMP-2 or in the presence of soluble BMP-2 (sBMP-2) and on biomimetic films in the absence of BMP-2 or in the presence of matrix-bound BMP-2 (bBMP-2). Images were taken after 1 day, three days and five days. Scale bar: 1mm.

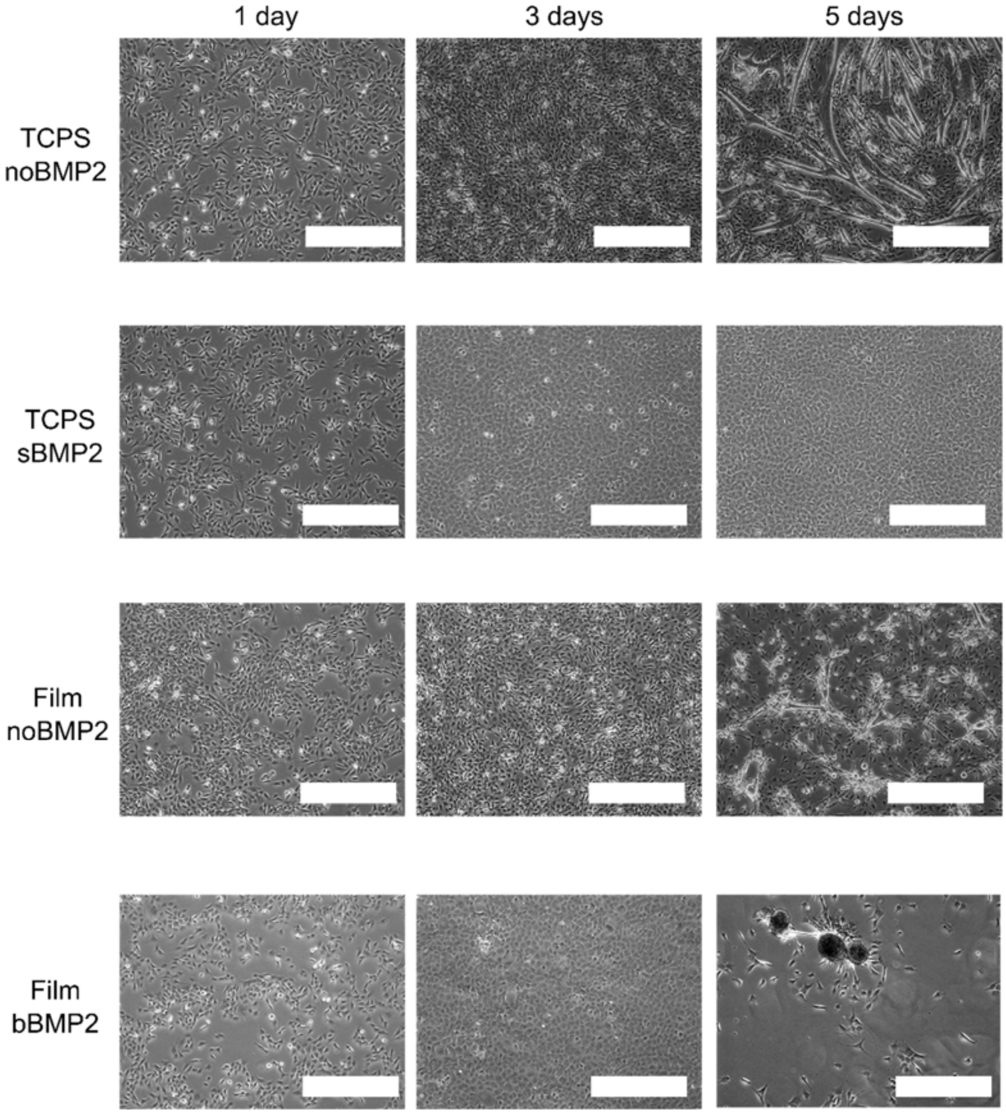

FIGURE SI2. Identification using the ENCODE database of muscle and bone-specific integrins and cadherins. (**A**) Percentage of expression of ITGA chains in muscle (top) or bone cells (bottom) were obtained by analyzing RNA sequencing data made for the ENCODE public research project. Pie charts illustrate the predominance of certain adhesion receptors in each cell type, especially for ITGA chains and cadherins. To highlight the specificity of the adhesion receptor repertoire in muscle versus bone cells, the ratio of muscle versus bone expression was calculated (**B**). Table (**C**) summarizes the highlighted adhesion receptor repertoire for each cell type. The underlined adhesion receptors were the most studied in the literature in this context of differentiation.

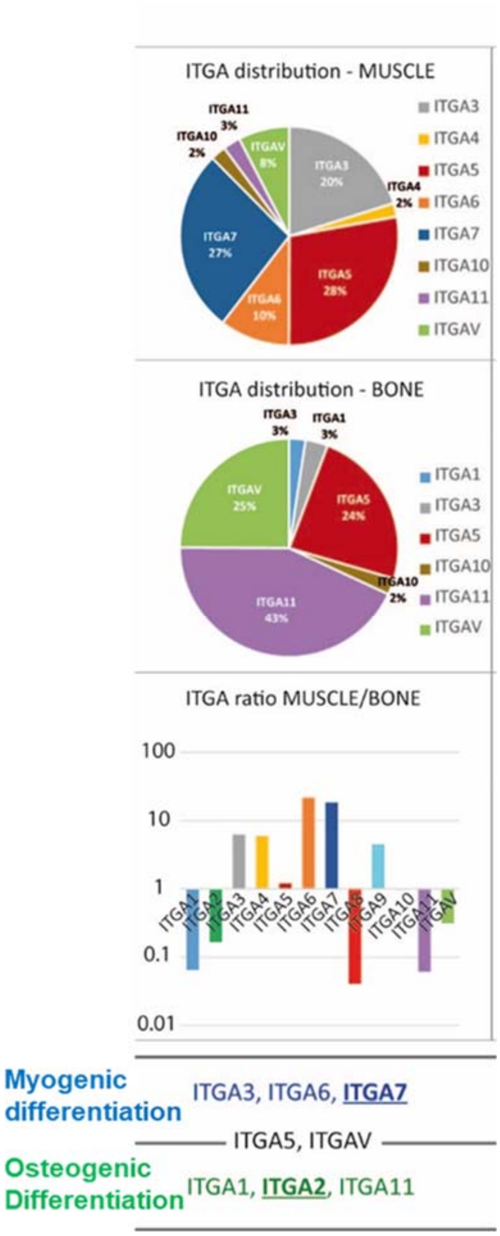

FIGURE SI3. Kinetics of gene expression for fibronectin-specific integrins (αV and α5) and of protein expression. Gene expression was quantified by RT-qPCR on TCPS without (blue) or with (green) sBMP-2 in solution (A). Protein expression was quantified by Western blot (A'). (A") Corresponding quantitative analysis of protein expression. Actin was taken as control for intensity normalization. Data are mean ± SD of three independent experiments.

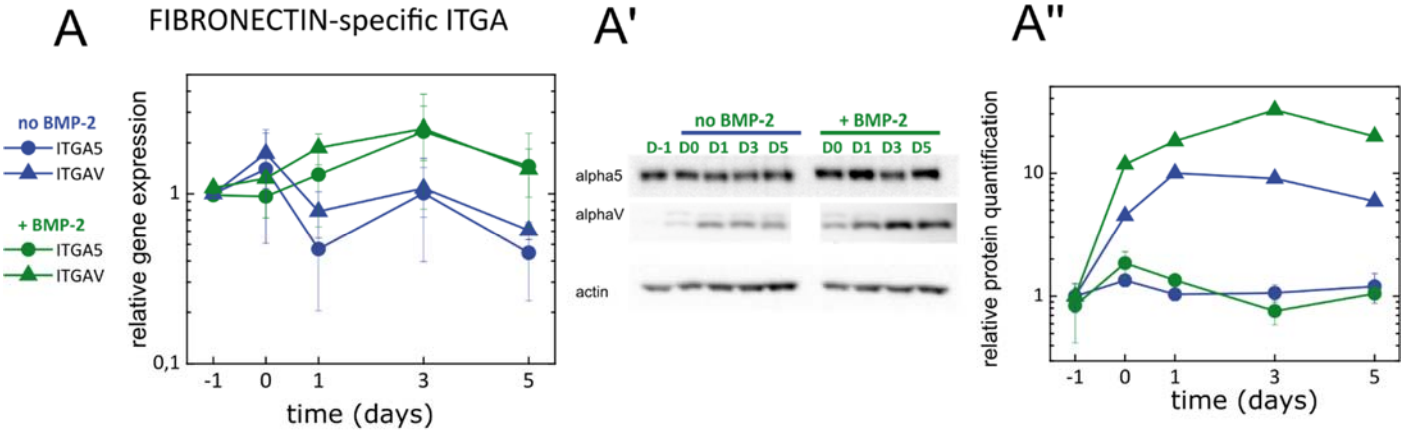

FIGURE SI4. Kinetics of gene expression for collagen-specific integrins. Gene expression was quantified by RT-qPCR on TCPS without (blue) or with (green) sBMP-2 in solution. Data are mean ± SD of three independent experiments.

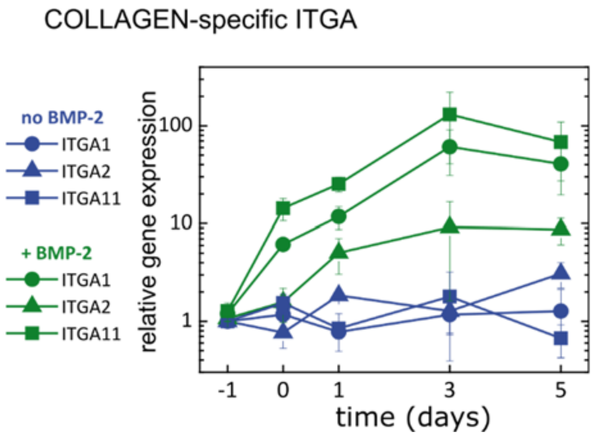

Fig. SI5. Efficiency of receptor silencing as measured by qPCR. The normalized expression of each gene was quantified after silencing, in comparison to the scramble condition.

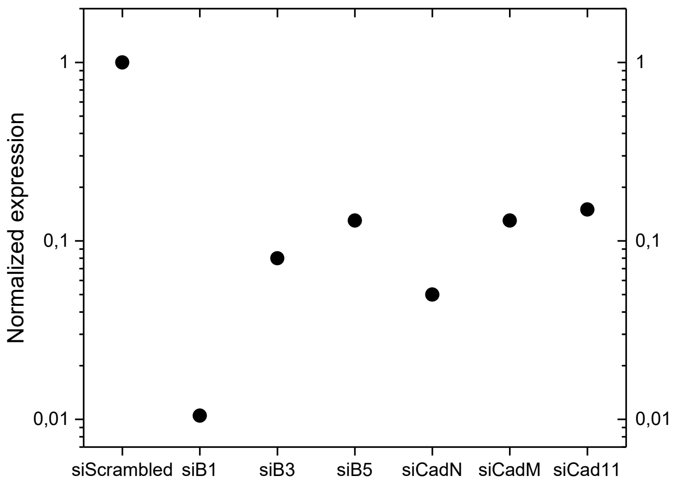

Supplement: Supplementary file 1 [file DataSheet1.PDF]
